# Perspectives on Neuromyelitis Optica Spectrum Disorders, the Narrative Medicine contribution to care

**DOI:** 10.1007/s10072-023-07146-4

**Published:** 2023-11-03

**Authors:** Massimo Filippi, Giovanna Borriello, Francesco Patti, Matilde Inglese, Maria Trojano, Fabiana Marinelli, Clara Chisari, Pietro Iaffaldano, Chiara Zanetta, Paola Chesi, Roberta Termini, Maria Giulia Marini

**Affiliations:** 1 IRCCS Ospedale San Raffaele, Unità di Neurologia e Neurofisiologia, Milan, Italy; 2 Centro di riferimento Regionale per la Sclerosi Multipla, Ospedale San Pietro Fatebenefratelli, Rome, Italy; 3 Dipartimento GF Ingrassia, Scienze Mediche e Chirurgiche e Tecnologie Avanzate, Università di Catania, Catania, Italy; 4 UOS Sclerosi Multipla, AOU Policlinico G Rodolico San Marco, Università di Catania, Catania, Italy; 5 Department of Neurology, Rehabilitation, Ophthalmology, Genetics, Maternal and Child Health (DINOGMI), University of Genoa, Genoa, Italy; 6 IRCCS Ospedale Policlinico San Martino, Genoa, Italy; 7 Department of Translational Biomedicine and Neurosciences, DiBraiN, University of Bari Aldo Moro, Bari, Italy; 8 ASL Frosinone, Ospedale Fabrizio Spaziani UOC Neurologia, Centro Sclerosi multipla, Frosinone, Italy; 9 Healthcare area, Fondazione ISTUD, Milano, Italy

**Keywords:** Illness experience, NMOSD, Narrative Medicine, Language analysis

## Abstract

**Background:**

This research aimed to investigate the experience of Neuromyelitis Optica Spectrum Disorders (NMOSD) by integrating the perspectives of patients, caregivers and clinicians through narrative-based medicine to provide new insights to improve care relationships.

**Methods:**

The research was conducted in the second half of 2022 and involved six Italian centres treating NMOSD and targeted adult patients, their caregivers and healthcare providers to collect the three points of view of living with or caring for this rare disease, still difficult to treat despite the pharmacological options. Narratives followed a structured outline according to the time: yesterday-today-tomorrow, to capture all disease phases.

**Results:**

Twenty-five patients diagnosed with NMOSD, ten caregivers and 13 healthcare providers participated in the research. Patients reported symptoms limiting their daily activities and strongly impacting their social dimension. We noticed improvements across disease duration, whilst the persistence of limitations was recurrent in patients with longer diagnoses.

Caregivers’ narratives mainly share experiences of their daily life changes, the burden of the caregiving role and the solutions identified, if any. Healthcare providers defined their role as a guide.

**Conclusion:**

Limitations in activities are prominent in the lives of people with NMOSD, along with fatigue. Family members are the weakest link in the chain and need information and support. Healthcare professionals are attentive to the helping dimension.

**Supplementary Information:**

The online version contains supplementary material available at 10.1007/s10072-023-07146-4.

## Introduction

Neuromyelitis optica (NMO) has recently been renamed to the term Neuromyelitis Optica Spectrum Disorders (NMOSD) to group a series of inflammatory diseases of the central nervous system (CNS) that primarily, though not exclusively, affect the optic nerve and spinal cord. NMOSD is a rare (1–2 people affected per 100,000 [1]) antibody-mediated disease of the central nervous system (CNS) [2]. Disease onset ranges between 4 and 88 years with a mean age of 39 years^2^. Women are disproportionately more often affected and, particularly in aquaporin-4-seropositive patients, female-to-male ratio can reach up to 10:1 [1, 2].

In most patients with NMOSD, autoantibodies against the astrocyte aquaporin-4 (AQP4) water channel are detectable, and patients typically suffer from recurrent attacks of severe optic neuritis or/and myelitis. In rarer cases, brainstem and brain involvement can occur. Patients also frequently suffer from pain, headache, depression, fatigue and sleep disorders. Despite treatment, attack recovery is often incomplete, and disease remission rarely occurs [3]. Thus, in relapsing NMOSD, which accounts for approximately 80–85% of cases, neurologic deficits frequently accumulate during the disease course [3].

There is no specific cure for NMOSD, but several treatments are available to control clinical manifestations and prevent relapse by reducing their frequency [2]: high-dose steroids (HDS) to treat acute attacks, plasma exchange in case of severe neurological deficits, thromboprophylaxis in non-ambulant patients with myelitis. Increasing knowledge of disease mechanisms has allowed the identification of new therapeutic targets for long-term relapse prevention treatment, such as monoclonal antibodies currently in evaluation in randomised clinical trials. Despite significant steps ahead in treating NMOSD, therapies improving regeneration and restoring functionality are still missing [3].

In addition to the clinical impact, this condition implies major lifestyle and psychological changes for the person affected and, in severe cases, caregivers and entire family units.

Narrative Medicine based on illness narratives [4] aims to integrate disease-centred and illness-centred, and sickness-centred approaches, which focus on individual experience and the social understanding of a specific condition [5]. In research, narrative-based medicine (NBM) suggests potential interventions for a particular condition and its care by integrating the point of view of all actors involved, including healthcare professionals and caregivers, with the twofold aim of mediating the patients’ context and understanding their perspective [6, 7]. The range of applications for NBM is from clinical practice to therapeutic path design, education, communication to increase awareness of different conditions, and research to investigate new elements to improve care services [8]. Whilst evidence-based medicine (EBM) concentrates on clinical features, NBM includes the personal experience in coping with distress [9]. Integrating EBM and NBM provides clinicians with tools to strengthen their clinical practice with narrative competencies [10, 11]. WHO recommends using narrative research to improve healthcare policies at the level of the individual, the health professional, the healthcare organisation and the healthcare system; collecting multiple perspectives is also encouraged [12].

The application of NBM to different diseases and conditions, including rare diseases, is increasing. Several studies investigating the physical, emotional and social impact of NMOSD using validated QoL questionnaires have been published [13–17], but none describes the impact of NMOSD from NBM point of view.

The Narrating Neuromyelitis Optica Spectrum Disorders project is the first experience in Italy where patients are given a voice and the opportunity to tell their daily challenges.

Through the application of Narrative Medicine, the ‘Narrating Neuromyelitis Optica Spectrum Disorders’ project aimed to conduct a listening activity through a systematic collection of narratives from three points of view.

Objectives of the research were the following:

- Understand the experience of people with NMOSD in daily living and repercussions on family, relational, work and social interactions, with a specific focus on the time of diagnosis and the experience with treatments.
- Integrate the experiences of family members of people living with NMOSD, and experts in the treatment of NMOSD, to understand their experience of relationship and care.

## Materials and methods

### Research design and setting

The, conducted online in 2022, involved six Italian public and university hospitals treating NMOSD in Italy:

1. IRCCS Ospedale San Raffaele, Unità di Neurologia e Neurofisiologia, Milan
2. Ospedale San Pietro Fatebenefratelli, Centro di riferimento Regionale per la Sclerosi Multipla, Rome
3. AOU Policlinico G Rodolico San Marco, UOS Sclerosi Multipla, University of Catania
4. Ospedale Policlinico San Martino, DINOGMI, University of Genoa
5. Department of Translational Biomedicine and Neurosciences - DiBraiN University of Bari Aldo Moro
6. ASL Frosinone, Ospedale Fabrizio Spaziani UOC Neurologia - Centro Sclerosi multipla, Frosinone

and the patients’ association, Italian Association of Multiple Sclerosis (AISM); Italian Association of NeuroMyelitis Optica (AINMO).

Referents from hospital centres and AISM composed the Steering Committee of the research. All methods and steps of the project, survey instruments and results that emerged from the analyses were shared within the committee.

## Data and narrative collection

A sociodemographic survey and three different narrative plots related to the illness experience were addressed to the three target groups: patients, caregivers and healthcare professionals (supplemental material provided) [18, 19].

The median Expanded Disability Status Scale (EDSS) was also used by treating physicians better to describe their patients’ clinical condition [20].

Narrative stimuli were used to invite patients to describe their experiences with NMOSD, from the first symptoms they noticed in the past, moving towards the present, to their perspectives and expectations on the future. The caregiver’s narrative plot included another section to collect their experiences with caregiving, whilst the healthcare providers narrative plot recorded information on their experiences during patient interactions.

Clinicians from the six medical centres, in 2022, directed the participants to the dedicated website https://www.medicinanarrativa.eu/narrare-la-neuromielite-ottica, in which, after being informed on the project design and purpose, and providing their informed consent, they could write their narratives. Data and narratives were collected anonymously through the Alchemer online survey platform [21]; at the end of data collection, raw data were downloaded as an Excel spreadsheet (Microsoft, Redmond, WA, USA).

## Data and narrative analysis

Researchers analysed the sociodemographic survey through descriptive statistics. Participants’ narratives were entered into the semantic evaluation software MAXQDA [22] for coding and analysis.

The texts were analysed through a qualitative-quantitative analysis. The narratives were analysed and clustered based on recurrences and main semantic groups and evaluated in their entirety and uniqueness using Narrative Medicine classifications. A comprehensive scenario of the experience of NMOSD from the three perspectives of patients, caregivers and healthcare professionals was obtained [23].

Researchers applied Kleinman’s classification, which distinguishes between disease-centred, illness-centred or sickness-centred narratives: specifically, disease narratives focus on the clinical evolution of a condition, using highly technical language, whereas illness narratives highlight individual emotional and relational experiences in an open and flowing narration; sickness narratives reflect society perception of a given condition.^6^ Overall evidence was reported in aggregate form.

The narratives were analysed considering: themes, language, narrative style, recurring words and use of metaphors in the relational and organisational aspects of the care pathways [23].

Researchers followed the Standards for Reporting Qualitative Research guidelines [24] in sharing the analysis results.

## Results

### Sociodemographic characteristics

Overall, 48 narratives from people with NMOSD (*n*=25), their caregivers (*n*=10) and their healthcare providers (*n*=13) were collected. The analysis was done by grouping patient, caregiver and physician narratives, respectively.

Amongst the 25 patient narratives collected, 92% of respondents were women, mean age of 50 years (range 29–71).

The sociodemographic characteristics of patients with NMOSD are shown in Table 1.

**Table 1:** Sociodemographic data of participating patients with NMOSD.

*Gender %(N)*	Female: 92% (23)	Male: 8% (2)					
*Age (range)*	Mean: 50 y (range: 29–71)						
*Place of residence %(N)*	North of Italy 40% (10)	Centre 28% (7)	South 32% (8)				
*Marital status %(N)*	Married/cohabitating 64% (16)	Single 20% (5)	Divorced 8% (2)	ND* 8% (2)			
*Education %(N)*	Secondary school second level 52% (13)	University degree 24% (6)	Secondary school first level 20% (5)	ND* 4% (1)			
*Occupation %(N)*	Employed 36% (9)	Invalid condition 20% (5)	Unemployed 16% (4)	Self-employed 8% (2)	Retired 8% (2)	Housewife 8% (2)	Not declared 4% (1)

An average of 4 years between the first symptoms to diagnosis corresponds to the start of the treatment; the final referral centre was also identified about another year after diagnosis.

## Findings from people with NMOSD: text analysis

Narratives followed a structured outline according to the time: in a diachronic fashion, yesterday-today-tomorrow, to picture all disease phases.

### Yesterday

This section was focused on the first signals that anticipated the diagnosis of NMOSD and the start of therapy, together with changes in daily life and relationships.

Terms used by patients during the narration of this phase highlighted active life (54%), other concomitant diseases (20%), being in good health (13%) and light-heartedness (13%).

Ninety-six percent of respondents described symptoms in detail. The average number of signs at onset was 2.9 (range 1–7).

**Fig. 1:**
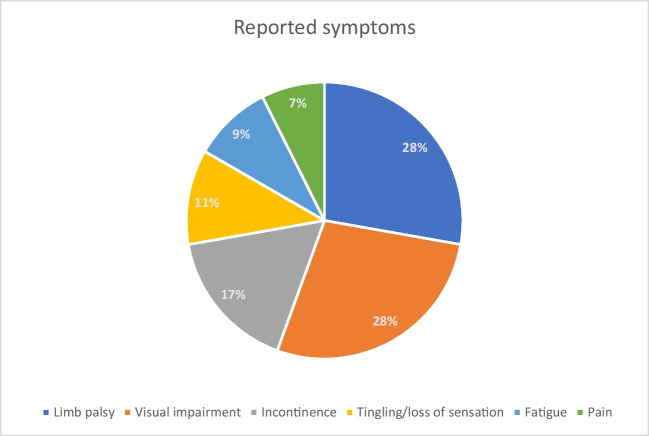
Most frequently reported symptoms.

Most cited symptoms are reported in Fig. 1.

In 28% of cases, stories told of initial misinterpretation of the disease leading to the diagnosis of multiple sclerosis, stress or visual problems.

In 20% of cases, female respondents experienced symptoms during pregnancy; in two cases, due to the onset of the disease; in the other three patients resulting in worsening symptoms after delivery.

Communication of diagnosis was most frequently a traumatic moment, followed by fear, confusion, sadness, difficulties and limitations, with a few positive reactions. Patients defined their bodies as blocked or limited by symptoms (64%); in 56% of the narratives, the theme of limitation experienced in everyday life was predominant.

In this first period, relationships with others were most frequently described as complex (56%); respondents did not feel understood, did not know how to relate with others and sometimes decided to isolate themselves. In 44% of cases, the desire at this stage was to resume one’s life and activities (28%) and regain autonomy (16%).

### ‘Today’s narratives’ (at the time of collection)

Cases of life limited by the disease continue to predominate (43%), followed by 39% narratives of improvement and 18% standing still situations. Cases of progress are present across all disease duration, whilst cases of persistence of limitations are more common amongst those diagnosed with NMOSD for more than 10 years. Acceptance is more frequent amongst those diagnosed within 5 years.

The topic of limitation and mental and physical fatigue remain prominent (64%) in people with NMOSD; however, positive feelings of well-being and improvement (44%) increase with reduced sadness and fear; confusion gives way to gratitude.

Daily activities comprehend home and family management and work. For people with NMOSD, the chance of continuing working is not just a necessity but remains a source in 28% of cases. Those who had to leave work aspired to resume working.

Relationships with others improve (57%), although 43% remain difficult. Close people are mostly represented by family members (60%), who share the burden of illness within 52% of cases. Definitions and descriptions of what NMOSD is reflect the difficulty of accepting a condition that is most frequently defined with terms such as ‘sneaky’, ‘monstrous’ or ‘unknown’, remarking the difficulty in being understood by others. However, descriptions of living with the disease are not rare.

In 44% of cases, treatments are recognised as adequate types and doses; 62% of respondents defined the relationship with physicians as positive.

‘Tomorrow’s’ expectations of people with NMOSD sway mainly between complete recovery (34%) and not getting worse (31%), followed by good quality of life and autonomy (16%) and plans for family and work life.

## Findings from caregivers of people with NMOSD

Ten caregivers participated in the project, equally divided between women and men, average age of 60 years. The majority are partners of people with NMOSD (60%), then children (20%) and sisters/brothers (20%).

Their narratives mainly tell of the limitations brought by NMOSD both for the person with the disease and for them, through descriptions of daily life changes, the burden of the caregiving role and, in some cases, the solutions identified.

As for patients, these stories also begin with active lives (46%) or defined as ‘normal’ (27%) and carefree (18%) before the onset of the debilitating symptoms; emotions and moods echo those of patients, especially in the sadness and fear felt at the beginning, but these are compounded by helplessness, present both about the past and the present, and fatigue, both physical and emotional, detected in the present. There is a positive experience of understanding and acceptance of the situation and the pride and admiration felt for their loved ones. The disease impacts caregivers’ daily living, decreasing from 88% in the beginning to 70% in the present.

NMOSD is predominantly described with negative terms (78%) and, in 22% of cases, with a lack of knowledge. Therapies are considered non-resolving (50%), although still necessary and effective (40%), sometimes burdensome (10%). Regardless of the results obtained from treatments, relationships with caring physicians are considered satisfactory by 62% of caregivers. For the future, too, there is correspondence with the expectations of people with NMOSD, with hope for a definitive cure (37%).

## Findings from healthcare providers

We received thirteen narratives from health care professionals, 85% represented by women, with an average age of 37 years (range 25–64). Seventy-seven percent of the respondents are neurological physicians, 15% are nurses and 8% are psychologists.

In describing their professional role through metaphor, images defining their role as a guide (helmsman, compass, expert travellers) in complexity (firefighter, strategist) prevail. Professionals sometimes feel they support people in their care (haven, shoulder to lean on).

Patients in care told about are primarily women (85%), with an average age of 43 years, in treatment at their centre for 4.9 years and diagnosed 4.8 years earlier. Their first symptoms appeared in the mean 6 years before. Thirty percent of these patients have comorbidities, and 54% have had episodes of recurrence of Neuromyelitis Optica.

The median Expanded Disability Status Scale [15] (EDSS) score is 3.0 (range 0–7).

At the first meeting with the patients, professionals were confronted with patients’ fear (54%) and suffering (31%). Most of the responding professionals who cared for the patients later communicated their diagnosis (38%). In subjects who received the diagnosis, the patient most frequently described confusion and trauma experienced.

Initially, empathy was predominant, but also discomfort because of the difficulties showed by patients, which in some cases made it difficult to initiate the treatment relationship. Currently, however, satisfaction and contentment with the results obtained prevail, and empathy remains, but, in some cases, discomfort remains, too, due to helplessness or concern about the progression of the disease. To date, cases of improvement in the condition of patients prevail (54%). Patients accept the situation and react, becoming more proactive and open. Caring for patients represents a challenge, an element of satisfaction and an opportunity for primary growth for these healthcare professionals.

Overall, the narration experience was positive in 73%, 89% and 90% of patients, caregivers and healthcare providers.

## Language analysis

Factual and didactic styles prevail in the narratives of patients living with NMOSD due to the descriptions of the treatment journey’s events, symptoms and stages.

Dramatic style is also present in some narratives, especially in the progression of the disease.

However, almost all the narratives (92%) made extensive and varied use of spontaneous metaphors, which were very expressive in rendering especially images of body changes, fatigue, suffering, the struggle with the disease and the upheaval it brought to lives. Metaphors most used by patients clustered by type are shown in Table 2, with some narrative fragments included in each cluster.

**Table 2:** Metaphors used by patients clustered by type.

Cluster of metaphors	Narrative fragments
Body changes and fatigue	My body was a stiff wood - My body, but not my brain, was totally shut down - Your body is constantly on strike - My leg was like dead - I had reduced myself to a wreck.
Suffering	I wanted it all to be a bad dream - Neuromyelitis is my cross, my recurring nightmare - My ordeal began again - I had to undergo this hell - This dark period.
Fighting the disease	I fight relentlessly; I never stop fighting - Finally, I knew what I had to fight with - Sometimes, I lose the willingness to fight.
Upheaval after diagnosis	Like a thunderbolt - He had received a big thrashing - My world fell apart - The communication of the diagnosis was a cold shower.
Resumption	I feel reborn - I want to take charge of my life again - At this canter, my world opened up again.
Living with the disease	Neuromyelitis is a travelling companion one is forced to live with - NMO is a new chapter in my life.
Disease as a ‘monster’.	Neuromyelitis is an ugly beast - My monster has been named - The rot has always been inside.
Visual impairment	Just a black abyss - I see the world as if it were an impressionist painting to which some Picasso brushstrokes are added.

The narrative style used by the family members and caregivers of people with NMOSD traces the factual and didactic style already identified in patients’ narratives for the descriptive part of the symptoms and the course of treatment. In 80% of the narratives, spontaneous metaphors mainly describe the daily struggle with illness, suffering and the ‘salvation’ of care.

Regarding healthcare professionals’ narratives, they used professional, didascalic language and less frequently used spontaneous metaphors, opposite to patients and family members. They were always sensitive to the dimension of aid for their patients.

## Narrative Medicine classifications

Applying proper classifications of NBM to qualitative and language analyses allows further interpretation of the main aspects of living with NMOSD.

Kleinman’s classification [6] of patient narratives shows the main aspects reflected in the narratives**,** *disease* indicating the frequency of descriptions of symptoms and therapies (44%); *illness*, implying opening to emotions (39%); *sickness*, i.e., the disease from a socio-cultural point of view reported in 19% of narratives, mainly concerning the difficulty in recognition of NMOSD, but also to the choice to hide the disease from others.

In emotions mentioned in the texts, fear and sadness prevail, along with confusion, but followed by trust, acceptance and gratitude, always in coexistence with fatigue, in line with the evolution of the treatment path. The topic of sadness and psychological distress is mentioned in 24% of the narratives, either as a loss of desire to do things and meet people or as the need for psychological support. The most recurrent coping elements (strategies to investigate reactions to the disease condition) are care (29%), the closeness of others (17%), physicians and acceptance of the state (both 14%). Limitations (52%) represent the most significant blocking element in the narratives, followed by lack of autonomy (22%) and disease progression (13%). These results show the importance of treatment for improving the quality of life of people with NMOSD and how much symptoms impact their lives.

Analysing caregivers’ narratives through the keys of Narrative Medicine, specifically Kleinman’s classification, the prevalence of *illness* appears (50%), i.e., intimate, emotional and family life experiences, complemented by the *disease*, i.e., symptoms and care (45%). The most frequent moods throughout the care path were fear, hope, helplessness, admiration, pride in loved ones, affection/love and tiredness. In this context, love for the family members is a resource, the main driving force despite fatigue.

Healthcare providers’ narratives through Kleinman’s classification confirmed the co-presence of the more clinical, treatment-related aspects of *disease* and the relational aspects with the emotional experiences of *illness.* On the other side, therapeutic possibilities and caring relationships were the primary resources for health providers as a source of satisfaction, gratitude and even admiration for their patients.

**Fig. 2:**
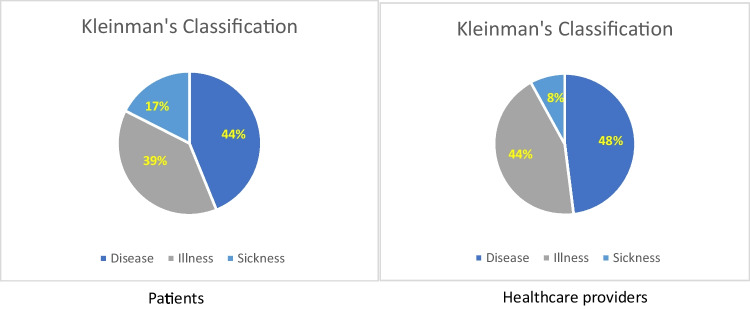
Kleinmann classification of narratives.

Comparing the three dimensions of Kleinman’s classification in patients and healthcare providers, whilst the disease dimension is almost equally present, the sickness dimension is more than doubly represented in patient narratives, indicating that healthcare providers perceive social aspects of NMOSD. However, closeness to their patients showed in the illness dimension. The results are shown in Fig. 2.

## Discussion

Narratives of Neuromyelitis Optica Spectrum Disorders integrated the three points of view of the person in care, family members and healthcare professionals, allowing the understanding of how they experience NMOSD. Narratives from patients show the trauma of losing their body’s functionality, or parts of it, previously fully active. Suddenly, a person has to learn to live with one-sided blindness or without mobility. Then, through an arduous but possible journey of awareness, patients learn to accept the limitations of their bodies. On the other hand, emotional and physical fatigue that makes acceptance difficult emerged. The terms ‘rare’ and ‘unknown’, revealing the fear of an unfamiliar condition and the lack of control, were recurrent. Patients struggled to share their feelings about being unrecognised as ill, with just a few physical symptoms. Thus, the reaction may be self-isolation or, as they regularly express in their narratives, ‘not weigh on’.

Most of the patients’ narratives came from women, most affected by this disease [2], sometimes living in solitude to hold the reins of the familiar nucleus, being mothers, wives and workers.

However, living with a rare disease also means that ‘maybe tomorrow research will discover the cure,’ an expectation we often find in the stories of patients and their families. To date, clinicians’ satisfaction with therapeutic advances of cures emerges from the narratives [3], enabling them to handle challenging situations. Patients accepted these treatments with gratitude, but the expectation of finding new options remains.

Narratives of family members who support people with NMOSD were mainly charged with affection and desire to be present and taken care of.

Family members are amongst the key players; they may be the weakest link and need practical and psychological support for the burden of caregiving.

The healthcare professional’s narratives used factual language, showing positivity, especially considering that they are also learning to cope with the condition that involves a complex, slow journey, which they deal with by integrating clinical care with relationship care. Patients’ fear and suffering at the first encounters are welcomed, understood and supported; time is given to find the proper care for everyone. Healthcare professionals are an excellent resource for this relapsing condition [3], which requires them to not just prescribe medications but to follow the evolution [25] of the disease in the person affected.

Considering the three different points of view, substantial alignment emerged from the narratives of patients and caregivers, whose main expectation is improvement in the physical condition and an effective cure in 50% and 64% of narratives, respectively. Eighty-five percent of professional narratives, instead, showed satisfaction with the therapeutic option and caring relationships offered, confirming what was already observed in other conditions [26].

Different emotions prevail in the narratives of the three groups considering limitations imposed by the disease, and patients felt significant limits of their condition. Contrarily, caregivers express a substantial burden.

A final consideration on the role of writing was an opportunity for people with NMOSD to share their experience and a tool to raise awareness for caregivers and health care professionals, who judge this novel tool as an opportunity to broaden their views on patient experiences and relationships. Narrative Medicine can be methodologically integrated into clinical practice.

## Limitations

Different numbers of narratives collected from patients (25), caregivers (10) and healthcare professionals (13) did not allow to truly triangulate the narratives to see the same story from three different points of view, nonetheless considering the rarity of NMOSD and the voluntary participation in the research, numbers obtained allowed to describe the three clusters of narratives and gather valuable insights for the treatment of this severe and impairing condition.

## Conclusions

The scientific community is making significant progress in the knowledge and treatment of NMOSD [27], and for this very reason integrating the clinical aspects of the disease, as it is defined in Narrative Medicine, into the daily, relational, intimate dimension of illness, with the final common goal to contribute to improving the quality of life of those living with NMOSD. From this experience with NM, we observed that for patients and caregivers, psychological support could be of help, especially in the early phases; and that young neurologists were captured by this novel tool that allowed them to broaden relationships with their patients, suggesting that educate health care professional on the potentiality of NM could contribute to better care of patients, as shown in previous experiences in other diseases [28–30].

### Supplementary information

ESM 1 (DOCX 101 kb)

ESM 2 (DOCX 102 kb)

ESM 3 (DOCX 101 kb)

## Data Availability

Original text narratives of the current research are available from the corresponding author upon reasonable request.

## Abbreviations

AQP4: Astrocyte aquaporin-4
CNS: Central nervous system
EBM: Evidence-based medicine
EDSS: Expanded Disability Status Scale
NBM: Narrative-based medicine
NMO: Neuromyelitis Optica
NMOSD: Neuromyelitis Optica Spectrum Disorder
