# Supplementary material for: Perspectives on Neuromyelitis Optica Spectrum Disorders, the Narrative Medicine contribution to care
Source: Neurol Sci. 2023 Nov 3;45(4):1589–97. doi: 10.1007/s10072-023-07146-4 (PMC10942930; doi:10.1007/s10072-023-07146-4)
Supplement: Supplementary file 1 — (DOCX 101 kb) [file 10072_2023_7146_MOESM1_ESM.docx]

**
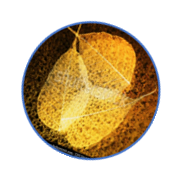
**

**Parallel chart for clinicians of patients with Neuromyelitis Optica Spectrum Disorders**

**NARRARE LA NEUROMIELITE OTTICA**

YESTERDAY

*When I first met him/her[…] My first impression on her/him[…]His/her body was[…]Diagnosis communication[…] I was[…] At home his/her relatives[…]*

*TODAY*

*She/he today[…] His/her body is[…]His daily routine is[…] She/he wants[…] The clinical treatment[…] When I meet her/him[…] Relationship with parenthal caregivers[…] Being his/her doctor is[…]*

*TOMORROW*

*When I think about his/her tomorrow[…] I would like to[…]*

NARRATIVE EXPERIENCE

*Telling my story was[…]*

**THANKS FOR YOUR ENERGY, THOUGHTS AND TIME. EVERY STORY COUNTS!**
