# Supplementary material for: Perspectives on Neuromyelitis Optica Spectrum Disorders, the Narrative Medicine contribution to care
Source: Neurol Sci. 2023 Nov 3;45(4):1589–97. doi: 10.1007/s10072-023-07146-4 (PMC10942930; doi:10.1007/s10072-023-07146-4)
Supplement: Supplementary file 2 — (DOCX 102 kb) [file 10072_2023_7146_MOESM2_ESM.docx]

**
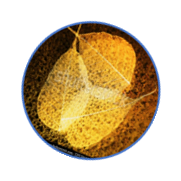
Illness plot for patients with Neuromyelitis Optica Spectrum Disorders**

**NARRATIVE MEDICINE TO INVESTIGATE THE QUALITY OF LIFE OF PATIENTS WITH NEUROMYELITIS OPTICA**

YESTERDAY

*Before Neuromielite Optica […] Suddenly […] People and medical centre I went to […] When I received the clinical diagnosis […] So I felt […] My body[…]* *(moving, looking around, talking, eating, drinking, was…) My daily routine was […] At home […] With others […] I would […]*

TODAY

*Today[…]I feel[…] My body… (moving my body, looking around, talking, eating, drinking is…) My daily routine is[…] At home[…] With others[…] Neuromyelitis Optica is[…] The treatment for Neuromyelitis Optica is[…] About medical doctors[…] I want[…]*

TOMORROW

*Tomorrow[…]I would like to[…]*

NARRATIVE EXPERIENCE

*Telling my story was…*

**THANK YOU FOR YOUR TIME, ENERGY AND THOUGHTS. EVERY STORY COUNTS.**
