# Supplementary material for: Perspectives on Neuromyelitis Optica Spectrum Disorders, the Narrative Medicine contribution to care
Source: Neurol Sci. 2023 Nov 3;45(4):1589–97. doi: 10.1007/s10072-023-07146-4 (PMC10942930; doi:10.1007/s10072-023-07146-4)
Supplement: Supplementary file 3 — (DOCX 101 kb) [file 10072_2023_7146_MOESM3_ESM.docx]

**
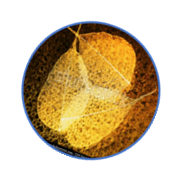
**

**Narrative plot for caregivers of patients with Neuromyelitis Optica Spectrum Disorders**

YESTERDAY

*Before Neuromyelitis Optica[…] Suddenly[…] People and medical centre we went to[…] When we received the clinical diagnosis[…] She/he was[…] So I was/I felt[…] His/her body[…]Her/his daily routine was[…] My daily routine was[…] At home[…] With others[…] She/He wanted[…] I wanted[…]*

TODAY

*She/he today[…] She/he feel[…] I feeL[…] Her/his body is[…]Her/his daily routine is[…]My daily routine is[…] At home[…] With others[…]Neuromyelitis Optica is[…] The treatment[…] About medical doctors[…] She/he wants[…] I want[…]*

TOMORROW

*Tomorrow[…] I would like to[…]*

NARRATIVE EXPERIENCE

*Telling my story was…*

**THANK YOU FOR YOUR TIME, ENERGY AND THOUGHTS. EVERY STORY COUNTS!**
